# DNA Data Bank of Japan (DDBJ) update report 2021

**DOI:** 10.1093/nar/gkab995

**Published:** 2021-11-09

**Authors:** Toshihisa Okido, Yuichi Kodama, Jun Mashima, Takehide Kosuge, Takatomo Fujisawa, Osamu Ogasawara

**Affiliations:** Bioinformation and DDBJ Center, National Institute of Genetics, Mishima, Shizuoka 411-8540, Japan; Bioinformation and DDBJ Center, National Institute of Genetics, Mishima, Shizuoka 411-8540, Japan; Bioinformation and DDBJ Center, National Institute of Genetics, Mishima, Shizuoka 411-8540, Japan; Bioinformation and DDBJ Center, National Institute of Genetics, Mishima, Shizuoka 411-8540, Japan; Bioinformation and DDBJ Center, National Institute of Genetics, Mishima, Shizuoka 411-8540, Japan; Bioinformation and DDBJ Center, National Institute of Genetics, Mishima, Shizuoka 411-8540, Japan

## Abstract

The Bioinformation and DDBJ (DNA Data Bank of Japan) Center (DDBJ Center; https://www.ddbj.nig.ac.jp) operates archival databases that collect nucleotide sequences, study and sample information, and distribute them without access restriction to progress life science research as a member of the International Nucleotide Sequence Database Collaboration (INSDC), in collaboration with the National Center for Biotechnology Information (NCBI) and the European Bioinformatics Institute. Besides the INSDC databases, the DDBJ Center also provides the Genomic Expression Archive for functional genomics data and the Japanese Genotype-phenotype Archive for human data requiring controlled access. Additionally, the DDBJ Center started a new public repository, MetaboBank, for experimental raw data and metadata from metabolomics research in October 2020. In response to the COVID-19 pandemic, the DDBJ Center openly shares SARS-CoV-2 genome sequences in collaboration with Shizuoka Prefecture and Keio University. The operation of DDBJ is based on the National Institute of Genetics (NIG) supercomputer, which is open for large-scale sequence data analysis for life science researchers. This paper reports recent updates on the archival databases and the services of DDBJ.

## INTRODUCTION

The DNA Data Bank of Japan (DDBJ) is a public database of nucleotide sequences established at the Bioinformation and DDBJ Center (DDBJ Center; https://www.ddbj.nig.ac.jp) of the National Institute of Genetics (NIG) (1). The DDBJ has been accepting annotated nucleotide sequences, issuing accession numbers, and distributing them in collaboration with GenBank at the National Center for Biotechnology Information (NCBI) (2) and the European Nucleotide Archive (ENA) at the European Bioinformatics Institute (EBI) (3) since 1987. This collaborative framework is known as the International Nucleotide Sequence Database Collaboration (INSDC) (4). As a node of INSDC, the DDBJ Center operates the DDBJ Sequence Read Archive (DRA) for raw sequencing data and alignment information generated by high-throughput sequencing platforms and analysis pipelines (5), the BioProject for study information, and the BioSample for sample information (1,6). This comprehensive biological data resource enriched with contextual study and sample information is available under the INSDC policy, which guarantees free and unrestricted access (7).

In addition to these INSDC databases, the DDBJ Center provides the Genomic Expression Archive (GEA) (8) for quantitative data from functional genomics experiments, such as gene expression and epigenetics, as the Gene Expression Omnibus at NCBI (9) and the ArrayExpress at EBI (10). Furthermore, the DDBJ Center services the controlled-access Japanese Genotype–phenotype Archive (JGA) to store and distribute the human genotype and phenotype data resulting from biomedical research in collaboration with the National Bioscience Database Center (NBDC, https://biosciencedbc.jp/en/) at the Japan Science and Technology Agency. Additionally, NBDC formulates the guidelines for sharing human data (https://humandbs.biosciencedbc.jp/en/guidelines) and hosts the Data Access Committee, which reviews applications for data submission and access to the JGA data in compliance with the guidelines (1,11). Furthermore, JGA collaborates with the major controlled-access databases, the database of Genotypes and Phenotypes (dbGaP) at NCBI (12), and the European Genome–phenome Archive (EGA) at EBI (13). Since September 2020, the JGA and NBDC systems have implemented the common account system so that users can conduct data submission and access applications on NBDC, upload and download the JGA data seamlessly (1).

In October 2020, the DDBJ Center launched a new public repository, MetaboBank (https://mb.ddbj.nig.ac.jp/search), for metabolomics data, in collaboration with the MetaboLights at EBI (14).

To automate submissions of genome sequences to DDBJ, the DDBJ Fast Annotation Submission Tool (DFAST), which is the service for annotating prokaryotic genomes and creating submission-ready DDBJ annotation and sequence files, has been developed (15).

DDBJ has collected and distributed genome sequences of bacterial type-strains covered by the Global Catalogue of Microorganisms (GCM) 10K type-strain sequencing project since 2020 as an international collaboration with the World Data Center for Microorganisms (WDCM) (16).

Because open sharing of SARS-CoV-2 genome sequences is critical to deal with the COVID-19 pandemic, INSDC released a statement asking the research community to share raw sequencing data and assembled sequences of SARS-CoV-2 genomes through INSDC (4). The DDBJ Center contributes to the open sharing by developing the analysis and submission systems of SARS-CoV-2 genome sequences in collaboration with Keio University and Shizuoka Prefecture in Japan. The collaboration is now called as the Japan COVID-19 Open Data Consortium.

Besides operating archival databases, the DDBJ Center provides the NIG supercomputer as a computational infrastructure for researchers to analyze biological data in Japan (1). The NIG supercomputer has expanded its storage system and calculation nodes to enable the analysis of ever-increasing sequencing data.

In this article, we report updates to the databases and services of the DDBJ Center. All resources are available at https://www.ddbj.nig.ac.jp, and the data are downloadable at ftp://ftp.ddbj.nig.ac.jp and https://ddbj.nig.ac.jp/public/.

## DDBJ ARCHIVAL DATABASES

### Data contents: unrestricted- and controlled-access databases

In 2020, the DDBJ accepted 6836 submissions of annotated nucleotide sequences, and 59.3% were submitted by Japanese research groups. The DDBJ has periodically released all public DDBJ/ENA/GenBank nucleotide sequence data in the flat-file format. The latest periodical release of June 2021 contains 2 830 321 188 sequences and 15 093 100 107 909 base pairs, and the DDBJ contributed 3.39% of the sequences and 2.23% of the base pairs.

Additionally, in 2020, the DRA accepted 59 583 runs of high-throughput sequencing data, and as of 25 August 2021, the DRA distributed 12.0 PB of sequencing data in the SRA (10.7 PB) and FASTQ (1.3 PB) formats. In 2020, the GEA accepted 84 submissions of data from functional genomics experiments. As of 25 August 2021, the GEA has provided 88 experiments at the ftp site (ftp://ftp.ddbj.nig.ac.jp/ddbj_database/gea). All public data of the archival databases are downloadable at https://ddbj.nig.ac.jp/public in addition to ftp://ftp.ddbj.nig.ac.jp.

Furthermore, in 2020, the JGA accepted 53 studies and 5776 samples submitted by Japanese research groups. As of 25 August 2021, the JGA has distributed 169 studies, 278 601 samples, and 293 TB of human data. Summaries of these studies are available to the public on the DDBJ Search (https://ddbj.nig.ac.jp/search) and the NBDC (https://humandbs.biosciencedbc.jp/en/data-use/all-researches) websites. Users must submit data usage requests to the NBDC to access individual-level data of these public studies. An overview of the statistics is available on our website (https://www.ddbj.nig.ac.jp/statistics/index-e.html).

### MetaboBank

The MetaboBank was launched in October 2020 as a public repository for experimental data and metadata of metabolomics research. Its record consists of raw and processed data files associated with detailed metadata describing the project design, analysis samples, experimental design and methods including instruments and measurement conditions. Each submission, called a project, is assigned an accession number with the prefix ‘MTBKS’ (for example, MTBKS1). As of 25 August 2021, the MetaboBank released 98 projects as available at https://mb.ddbj.nig.ac.jp/search.

### GCM 10K type-strain sequencing project

The Global Catalogue of Microorganisms (GCM) 10K type-strain sequencing project was organized by an international collaboration comprising the WDCM, culture collections, and the International Journal of Systematic and Evolutionary Microbiology(IJSEM) toward free access to standard genome sequencing and annotation services for microbial researchers (16). In this project, DDBJ is in charge of accepting and distributing genome sequences with annotation. Additionally, WDCM conducts genome sequencing of type strains, annotating the sequences using DFAST, and submitting them to DDBJ (17). As a result, DDBJ accepted and distributed 44 569 genome sequences of 690 type-strains available under the BioProject PRJDB9057.

### Open sharing of SARS-CoV-2 genome sequence

SARS-CoV-2 genome data should be freely available for everybody to overcome the COVID-19 pandemic. However, a large portion of SARS-CoV-2 genome data are registered to GISAID (https://www.gisaid.org/) without data registration to INSDC (4). GISAID does not allow bulk data download and lacks the functionality to deposit raw data. To complement the situation, INSDC encourages the scientific community to submit SARS-CoV-2 sequences to INSDC databases (https://www.insdc.org/sites/insdc.org/files/documents/INSDC_Statement_on_SARS-CoV-2_sequence_data_sharing_during_COVID-19.pdf) and GISAID. Toward the open sharing of SARS-CoV-2 genomic data in Japan, we have started the Japan COVID-19 Open Data Consortium.

**Figure 1.**
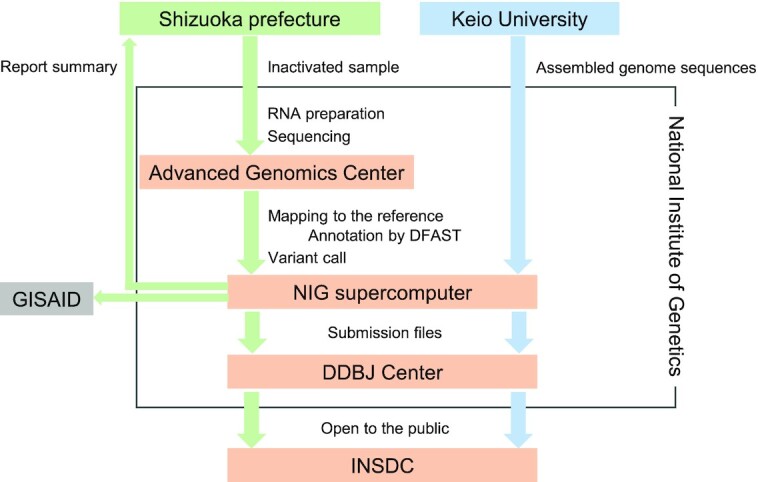
Overview of genome sequencing, mapping reads, annotation, and registration to INSDC for SARS-CoV-2 samples. In the case of Shizuoka Prefecture, after the National Institute of Genetics (NIG) receives inactive virus samples from Shizuoka Prefecture, NIG conducts several processes: genome sequencing at the Advanced Genomic Center, mapping raw reads to the reference, annotation by DFAST, and variant calls on the NIG supercomputer. After completion of sequencing and computational analysis, NIG reports the summary of the virus genome characteristics to Shizuoka Prefecture. Finally, the virus genome sequences are submitted to both GISAID and DDBJ. In the case of Keio University, sequencing and mapping are performed at Keio University.

NIG and the prefectural government of Shizuoka, where NIG is located, have cooperated in the molecular epidemiological investigation since April 2020 (https://www.nig.ac.jp/nig/2021/05/information/info20210430.html). NIG conducts next-generation sequencing of the virus samples collected by Shizuoka Prefecture, performs mapping of the raw sequencing data to the reference genome from Wuhan (NCBI RefSeq NC_045512), annotates the genomes by DFAST using VADR of NCBI (18), calls variants, and registers the annotated virus genome sequences to DDBJ (Figure 1). NIG reports the summary of virus genome characteristics to Shizuoka Prefecture for genome surveillance. As the first release of this cooperation, 47 virus genome sequences are available at INSDC under the accession numbers BS001145–BS001191. Additionally, the SARS-CoV-2 genome sequences are registered to both DDBJ and GISAID.

Another collaboration is with Keio University; NIG annotates the genome sequences of SARS-CoV-2 sampled, sequenced, and mapped at Keio University (Figure 1). As an initial phase of this collaboration, 452 genome sequences are available at INSDC under the accession numbers BS000685–BS001136. These activities represent uncommon inter-municipal and academic collaboration, which we wish to expand to other institutes or organizations collecting SARS-CoV-2 samples.

## DDBJ SYSTEM UPDATE

### Services for submitting biological data

The DDBJ Fast Annotation Submission Tool (DFAST, https://dfast.nig.ac.jp) was developed to realize automated annotation and submission of prokaryotic genomes (14). To improve the quality of the genome annotation, the DFAST also verifies the taxonomic assignment of the submitted genome by calculating the Average Nucleotide Identity (ANI) with reference genomes. In 2020, 85.8% (1806/2105) of the prokaryote submission were processed by the DFAST, showing 2.2-fold increase compared to 38.4% (768/1998) in 2019. In order to meet the increasing demand for the automated annotation service, the average processing time was shortened by half by parallelizing the job processes.

### The NIG supercomputer

The NIG supercomputer is used as a computational infrastructure to construct the archival databases, including INSDC, and is also provided to domestic researchers for research and education in life science (1). However, since the supercomputer system is subject to export control regulations under the Foreign Exchange and Foreign Trade Act, overseas users need to be collaborators of Japanese life science researchers to use the supercomputer.

The NIG supercomputer was installed in March 2019. The storage system consists of two parts: one is for archiving data of the databases (12.9 PB disk and 15 PB tape), and the other is for storing users’ data for analysis (16.8 PB, 3 PB was added in March 2021). The computation system consists of a general-purpose distributed memory cluster system and large memory computers for *de novo* assembly (80 CPU cores, 10 units of 3 TB main memory, and 1 unit of 12 TB main memory with 288 CPU cores). The general-purpose cluster system consists of 232 compute nodes with 14 336 CPU cores (345 TFlops of computing power) and a GPU (NVIDIA Tesla V100 SXM2) with 499 TFlops of computing performance. About one-third of the computing power is dedicated to constructing archival databases, reflecting an increasing demand for personal genome analysis since late 2020. About half of the remaining power has been used for analyzing personal genomics data requiring controlled access.

## FUTURE DIRECTION

The DDBJ Center contributes to the molecular epidemiological survey of SARS-CoV-2 virus by serving the computational power and open data sharing in compliance with the INSDC policy. Furthermore, we expand this collaboration model with Shizuoka Prefecture, Keio University, and other institutions and local governments to help overcome the COVID-19 pandemic in Japan.

The number of nucleotide sequence submissions to the databases is growing due to decreasing cost of sequencing technologies. We will implement DFAST with the submission functionality used in the BioProject, BioSample, DRA and GEA submission systems to further automate prokaryotic genome submissions. This integration allows the submitters to link BioProject and BioSample data more easily with DFAST data. Additionally, to reduce the cost for sequence submissions, we will also implement validation tools for annotation and sequence files so that submitters can correct data by themselves before submitting them to DDBJ.

## References

[1] Fukuda A. , Kodama Y., Mashima J., Fujisawa T., Ogasawara O. DDBJ update: streamlining submission and access of human data. Nucleic Acids Res. 2021; 49:D71–D75. 33156332 10.1093/nar/gkaa982 PMC7779041

[2] Sayers E.W. , Cavanaugh M., Clark K., Pruitt K.D., Schoch C.L., Sherry S.T., Karsch-Mizrachi I. GenBank. Nucleic Acids Res. 2021; 49:D92–D96. 33196830 10.1093/nar/gkaa1023 PMC7778897

[3] Harrison P.W. , Ahamed A., Aslam R., Alako B.T.F., Burgin J., Buso N., Courtot M., Fan J., Gupta D., Haseeb M. et al. The European nucleotide archive in 2020. Nucleic Acids Res. 2021; 49:D82–D85. 33175160 10.1093/nar/gkaa1028 PMC7778925

[4] Arita M. , Karsch-Mizrachi I., Cochrane G. The international nucleotide sequence database collaboration. Nucleic Acids Res. 2021; 49:D121–D124. 33166387 10.1093/nar/gkaa967 PMC7778961

[5] Kodama Y. , Shumway M., Leinonen R. The sequence read archive: explosive growth of sequencing data. Nucleic Acids Res. 2012; 40:D54–D56. 22009675 10.1093/nar/gkr854 PMC3245110

[6] Federhen S. , Clark K., Barrett T., Parkinson H., Ostell J., Kodama Y., Mashima J., Nakamura Y., Cochrane G., Karsch-Mizrachi I. Toward richer metadata for microbial sequences: replacing strain-level NCBI taxonomy taxids with BioProject, BioSample and Assembly records. Stand. Genomic. Sci. 2015; 9:1275–1277. 10.4056/sigs.4851102 PMC4149001 25197497

[7] Brunak S. , Danchin A., Hattori M., Nakamura H., Shinozaki K., Matise T., Preuss D Nucleotide sequence database policies. Science. 2002; 298:1333. 10.1126/science.298.5597.1333b 12436968

[8] Kodama Y. , Mashima J., Kosuge T., Ogasawara O. DDBJ update: the Genomic Expression Archive (GEA) for functional genomics data. Nucleic Acids Res. 2019; 47:D69–D73. 30357349 10.1093/nar/gky1002 PMC6323915

[9] Clough E. , Barrett T. The gene expression omnibus database. Methods Mol. Biol. 2016; 1418:93–110. 27008011 10.1007/978-1-4939-3578-9_5 PMC4944384

[10] Athar A. , Füllgrabe A., George N., Iqbal H., Huerta L., Ali A., Snow C., Fonseca N.A., Petryszak R., Papatheodorou I. et al. ArrayExpress update - from bulk to single-cell expression data. Nucleic Acids Res. 2019; 47:D711–D715. 30357387 10.1093/nar/gky964 PMC6323929

[11] Kodama Y. , Mashima J., Kosuge T., Katayama T., Fujisawa T., Kaminuma E., Ogasawara O., Okubo K., Takagi T., Nakamura Y. The DDBJ Japanese genotype-phenotype archive for genetic and phenotypic human data. Nucleic Acids Res. 2015; 43:D18–D22. 25477381 10.1093/nar/gku1120 PMC4383935

[12] Tryka K.A. , Hao L., Sturcke A., Jin Y., Wang Z.Y., Ziyabari L., Lee M., Popova N., Sharopova N., Kimura M. et al. NCBI’s database of genotypes and phenotypes: dbGaP. Nucleic Acids Res. 2014; 42:D975–D979. 24297256 10.1093/nar/gkt1211 PMC3965052

[13] Lappalainen I. , Almeida-King J., Kumanduri V., Senf A., Spalding J.D., Ur-Rehman S., Saunders G., Kandasamy J., Caccamo M., Leinonen R. et al. The European genome-phenome archive of human data consented for biomedical research. Nat. Genet. 2015; 47:692–695. 26111507 10.1038/ng.3312 PMC5426533

[14] Haug K. , Cochrane K., Nainala V.C., Williams M., Chang J., Jayaseelan K.V., O’Donovan C MetaboLights: a resource evolving in response to the needs of its scientific community. Nucleic Acids Res. 2020; 48:D440–D444. 31691833 10.1093/nar/gkz1019 PMC7145518

[15] Tanizawa Y. , Fujisawa T., Arita M., Nakamura Y. Generating publication-ready prokaryotic genome annotations with DFAST. Methods Mol. Biol. 2019; 1962:215–226. 31020563 10.1007/978-1-4939-9173-0_13

[16] Wu L. , Ma J. The Global Catalogue of Microorganisms (GCM) 10K type strain sequencing project: providing services to taxonomists for standard genome sequencing and annotation. Int. J. Syst. Evol. Microbiol. 2019; 69:895–898. 30832757 10.1099/ijsem.0.003276

[17] Shi W. , Sun Q., Fan G., Sugawara H., Ohkuma M., Itoh T., Zhou Y., Cai M., Kim S.G., Lee J.S. et al. gcType: a high-quality type strain genome database for microbial phylogenetic and functional research. Nucleic Acids Res. 2021; 49:D694–D705. 33119759 10.1093/nar/gkaa957 PMC7778895

[18] Schäffer A.A. , Hatcher E.L., Yankie L., Shonkwiler L., Brister J.R., Karsch-Mizrachi I., Nawrocki E.P. VADR: validation and annotation of virus sequence submissions to GenBank. BMC Bioinform. 2020; 21:211. 10.1186/s12859-020-3537-3 PMC7245624 32448124

